# Laparoscopic resection of a descending colon tumor with right-sided fixation of the sigmoid colon: a case report

**DOI:** 10.1186/s40792-024-02004-7

**Published:** 2024-08-30

**Authors:** Shinya Ohno, Yukimasa Nagata, Tatsuki Kawahara, Yusuke Nonomura, Reo Tachikawa, Tomohito Shinoda, Kakeru Tawada, Aiko Ikawa, Bun Sano

**Affiliations:** Department of Surgery, Takayama Red Cross Hospital, 3-11 Tenmanmachi, Takayama, Gifu, 506-0025 Japan

**Keywords:** Intestinal malrotation, Laparoscopic surgery, Symmetrical inferior mesenteric artery

## Abstract

**Background:**

Intestinal malrotation is a condition in which the process of counterclockwise rotation and fixation to the peritoneum and retroperitoneum during fetal life is incomplete. In adults, it is generally asymptomatic and is often discovered incidentally. We report a case of laparoscopic partial resection of the descending colon for a tumor of the descending colon with a rare form of intestinal malrotation in which the inferior mesenteric artery ran symmetrically and the sigmoid colon was fixed to the dorsal cecum and right-sided retroperitoneum.

**Case presentation:**

A 75-year-old man was referred to our department of internal medicine due to a positive fecal occult blood test. Lower endoscopy revealed a laterally spreading tumor in the descending colon, and endoscopic submucosal dissection was attempted; however, this procedure was difficult, and the patient was referred to our department for surgical treatment. Contrast-enhanced computed tomography revealed that the endoscopic clip was located in the descending colon on the right side, the inferior mesenteric artery was symmetrical, and the sigmoid colon was located on both the right and dorsal sides of the cecum. Laparoscopic ileocecum and sigmoid colon mobilization was performed from the left side of the patient. After the completion of sigmoid colon mobilization, which returned the sigmoid colon and descending colon to anatomical normalcy, laparoscopic partial resection of the descending colon was performed. Based on the results of a histopathological examination, a granular type of laterally spreading tumor was diagnosed. The patient was discharged uneventfully on postoperative day 8.

**Conclusions:**

Detailed preoperative imaging and surgical simulation are necessary for abdominal surgery involving intestinal malrotation.

## Background

The embryonic gastrointestinal tract is divided into the foregut, midgut, and hindgut, and the midgut rotates 270° counterclockwise around the axis of the superior mesenteric artery (SMA), and is fixed to the peritoneum and retroperitoneum. Intestinal malrotation is defined as a condition in which this process is incomplete [1, 2]. While most patients with intestinal malrotation develop gastrointestinal symptoms in childhood, intestinal malrotation in adults is generally asymptomatic, and is often detected incidentally [3, 4]. Right-sided fixation of the sigmoid colon is a rare anatomic condition in which the sigmoid colon is fixed to the right posterior abdominal wall and is often associated with an extra-long sigmoid colon and various types of intestinal malrotation [5]. We report a case of laparoscopic partial resection of the descending colon for a tumor of the descending colon with a rare form of intestinal malrotation in which the inferior mesenteric artery (IMA) ran symmetrically, and the sigmoid colon was fixed to the dorsal cecum and right-sided retroperitoneum.

## Case presentation

A 75-year-old man was referred to our department of internal medicine for a positive fecal occult blood test. Lower endoscopy revealed a laterally spreading tumor (LST) in the descending colon, and endoscopic submucosal dissection (ESD) was attempted. However, this procedure was difficult to perform, so the patient was referred to our department for surgical treatment. Ten years previously, the patient had undergone right inguinal hernia surgery via an anterior approach. He was not taking any medication.

**Fig. 1:**
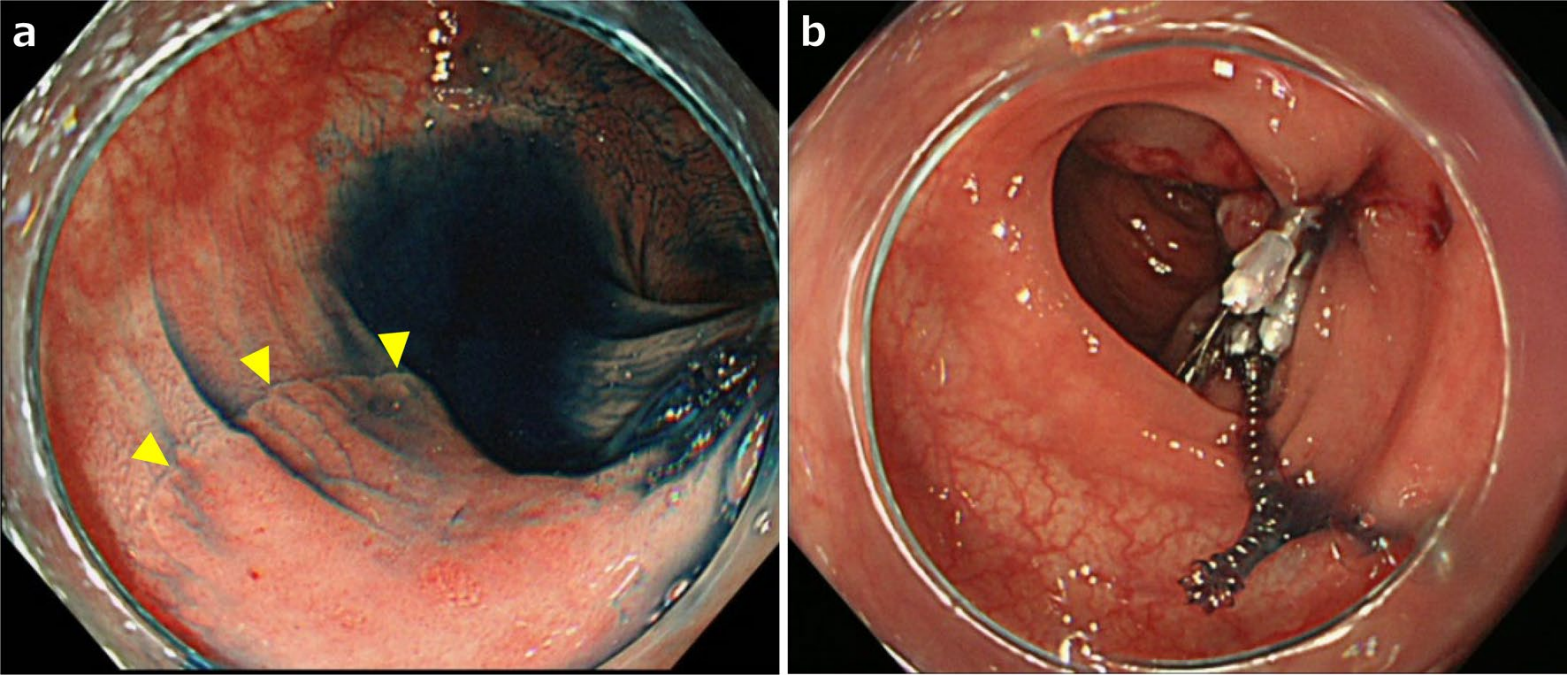
Upper endoscopy image. **a** Laterally spreading tumor in the descending colon (yellow arrowheads). **b** Clipping of the endoscopic submucosal dissection section

**Fig. 2:**
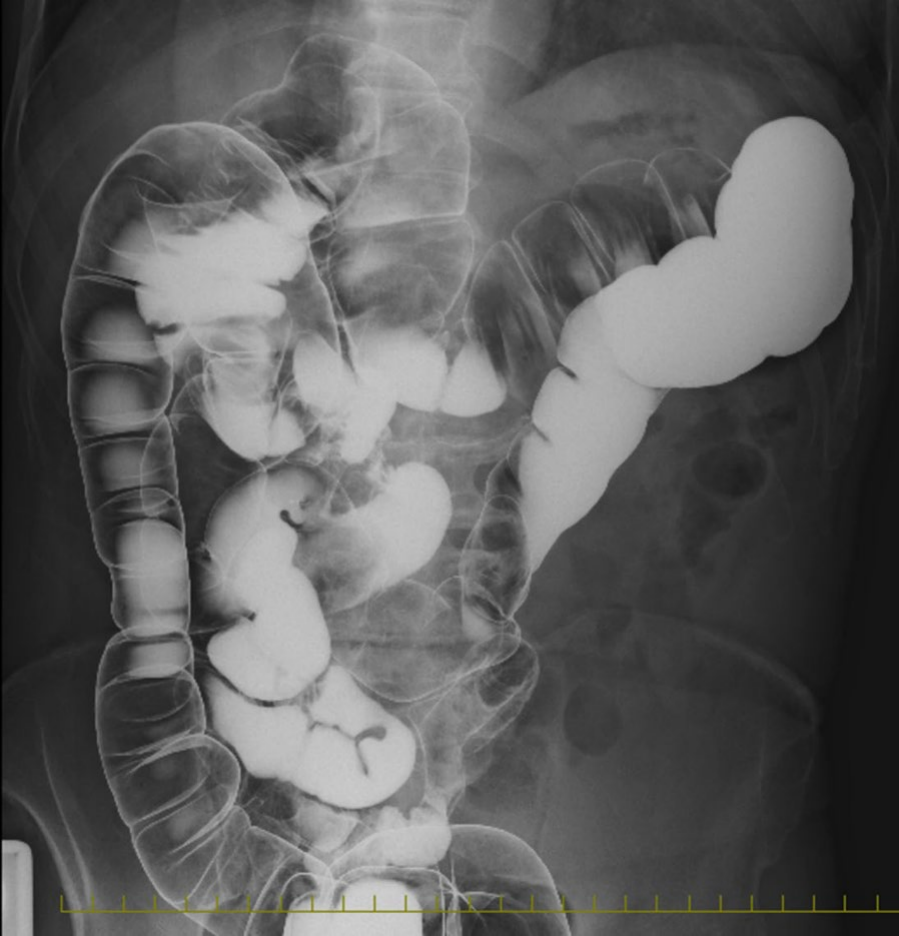
Enema examination. The descending colon to the sigmoid colon runs on the right side

**Fig. 3:**
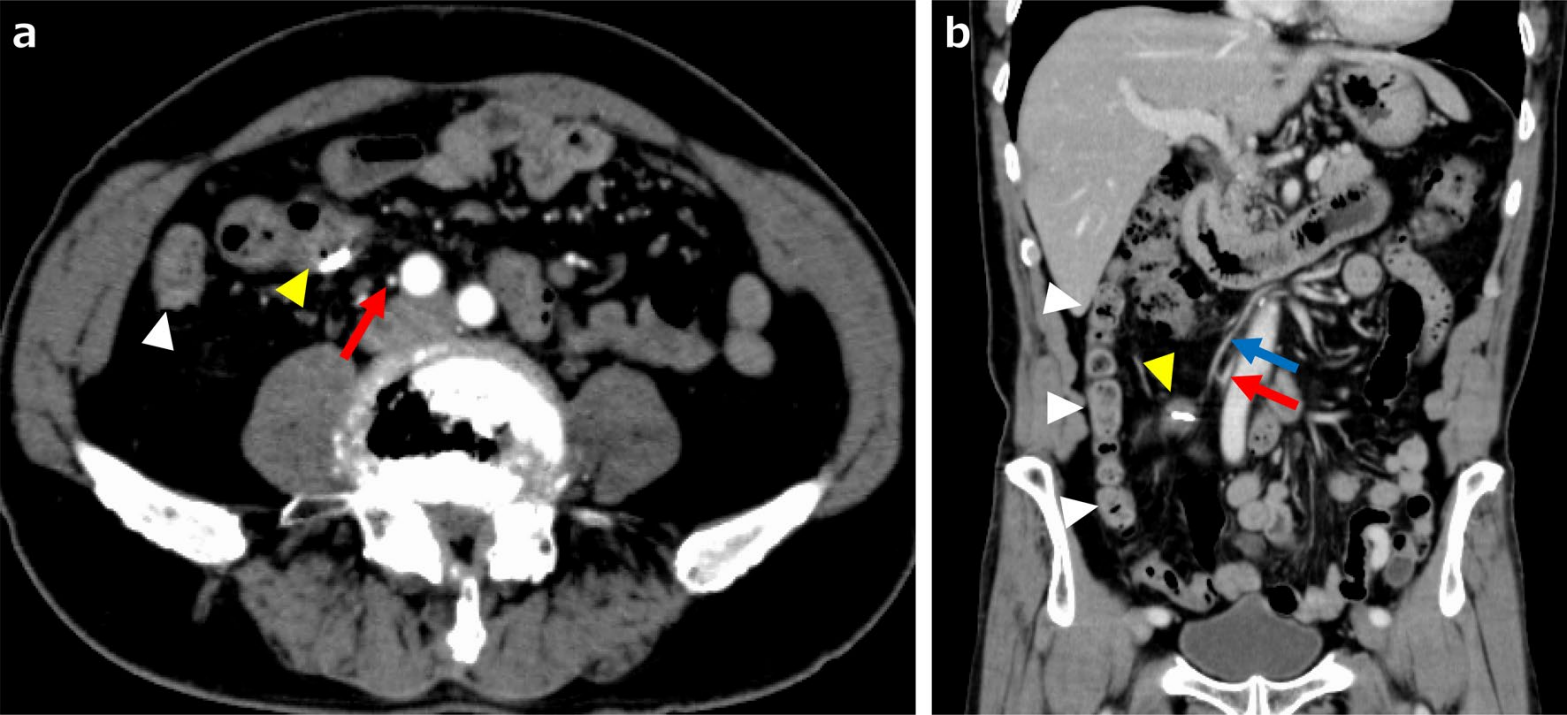
Contrast-enhanced computed tomography (CT) images. **a** The axial CT image revealed that the clip was located in the descending colon on the right side (yellow arrowhead), the IMA ran symmetrically (red arrow), and the sigmoid colon was located on the right side (white arrowhead). **b** A sagittal CT image revealing the clip in the right-sided descending colon (yellow arrowhead) and right-sided fixed sigmoid colon (white arrowhead) and symmetrical runs of the IMA (red arrow) and IMV (blue arrow)

**Fig. 4:**
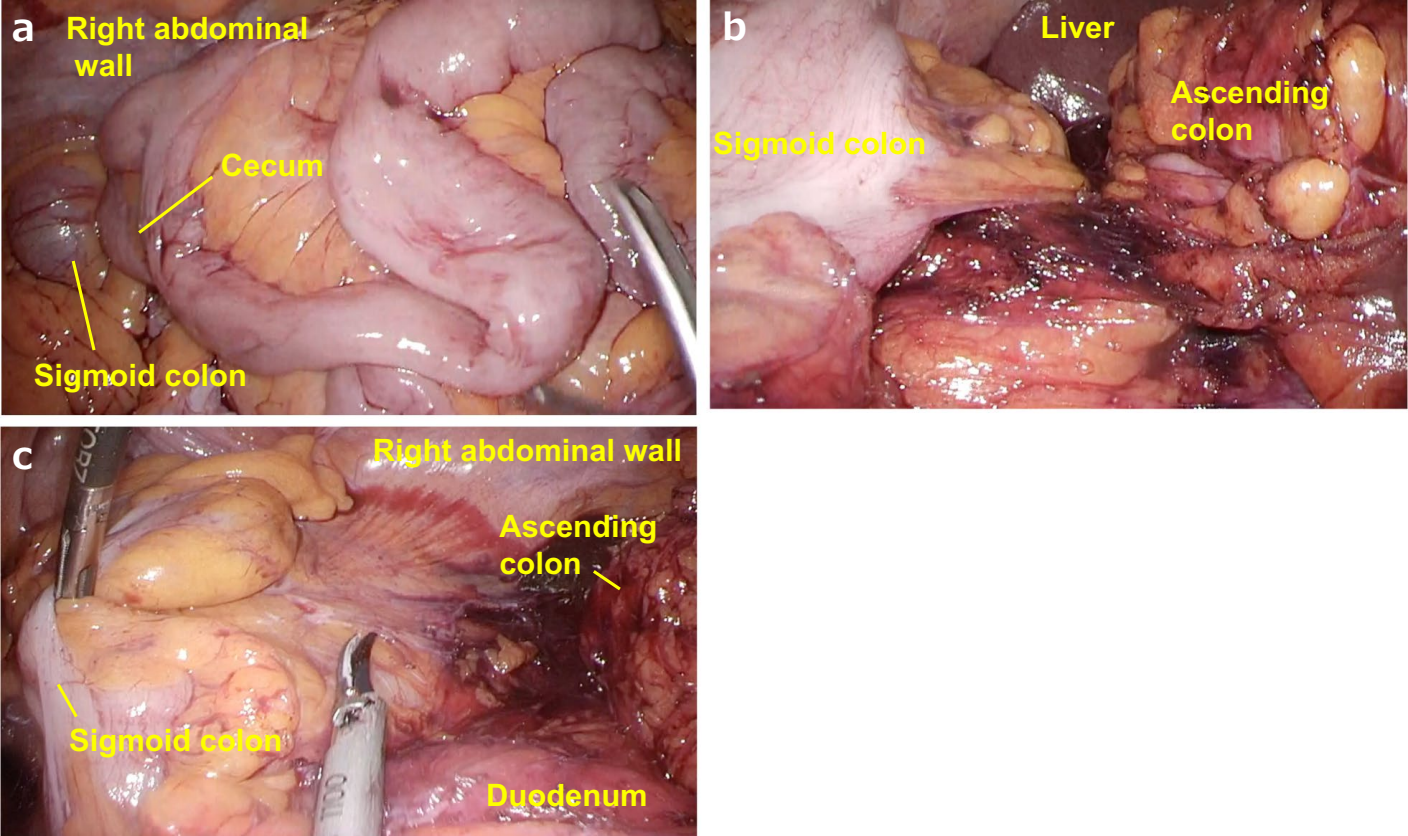
Intraoperative findings. The sigmoid colon was located on the right and dorsal sides of the ileocecum (**a**). After the ileocecum mobilization was performed, the sigmoid colon was fixed to the hepatic flexure (**b**) and right retroperitoneum (**c**)

A physical examination revealed the following: body weight, 67.0 kg; height, 169 cm; and body mass index, 23.5 kg/m^2^. There were no obvious abnormal findings on blood tests and his levels of carcinoembryonic antigen and carbohydrate antigen 19–9 (tumor markers) were within the normal range. Lower endoscopy revealed a 30-mm LST in the descending colon (Fig. 1A). ESD was attempted but incomplete due to surgical difficulties, and the ESD section was clipped (Fig. 1B). An enema examination revealed that the descending colon to the sigmoid colon ran on the right side (Fig. 2). Contrast-enhanced computed tomography (CECT) revealed that the endoscopic clip was located in the descending colon on the right side, the IMA was symmetrical, and the sigmoid colon was located on both the right and dorsal sides of the cecum (Fig. 3A, B). We diagnosed the patient with LST in the descending colon with incomplete intestinal malrotation, and laparoscopic partial resection of the descending colon was performed. Intraoperative findings revealed that the sigmoid colon was located on the right and dorsal sides of the ileocecum and was fixed to the hepatic flexure and right retroperitoneum (Fig. 4A, B, C). Laparoscopic ileocecum and sigmoid colon mobilization was performed from the left side of the patient. First, the ileocecum located ventral to the sigmoid colon was detached from the sigmoid colon from the caudal side, and after the detachment of the fixation to the right abdominal wall and the completion of sigmoid colon mobilization, which returned the sigmoid colon and descending colon to anatomical normalcy, laparoscopic partial resection of the descending colon was performed from the right side of the patient. Operative time was 219 min and the estimated blood loss was 10 mL. The histopathological diagnosis was LST granular type, 20 × 15 mm tubular adenoma with severe atypia, high grade, and pN0. The patient started oral intake on postoperative day (POD) 3 and was discharged uneventfully on POD 8. The patient progressed without recurrence one year after the operation.

## Discussion

Intestinal malrotation is an abnormality in the embryological development of the gastrointestinal tract. By the fourth week, the gastrointestinal tract is formed into an endoderm-lined tube. The vascular pedicle develops in the fifth week, and the gastrointestinal tube is divided into the foregut, midgut, and hindgut. The SMA supplies blood to the midgut, which rapidly expands its loop to form the jejunum, ileum, and colon up to the splenic flexure.

The rotation of intestinal development is divided into three stages, with stage 1 occurring at weeks 5 to 10 and involving extrusion of the midgut out of the embryonic cavity, 90° counterclockwise rotation and the return of the midgut to the fetal abdomen. Midgut nonrotation results from the cessation of development at stage 1. Stage 2 occurs at week 11 and involves further counterclockwise rotation in the abdominal cavity to complete a 270° rotation. This rotation positions the “C” loop of the duodenum posterior to the SMA, forming the ascending colon on the right, the transverse colon upward, and the descending colon on the left. Anomalies in this phase include malrotation and reversed rotation. Stage 3 is the descent of the cecum into the right lower abdomen and fixation of the mesentery, which lasts from week 11 to full term. Abnormalities in this stage cause an unfixed duodenum, a mobile cecum, an unfixed cecum, and an unattached mesentery of the small intestine [2, 6–9].

Previous reports have described right-sided sigmoid colon was caused by extra-long redundancy of the sigmoid colon and/or midgut malrotation [5], fixation anomalies [10], or secondary rotation of the colon during embryogenesis [11], but no report has mentioned a specific embryological mechanism of a right-sided sigmoid colon located on the right and dorsal sides of the cecum.

**Fig. 5:**
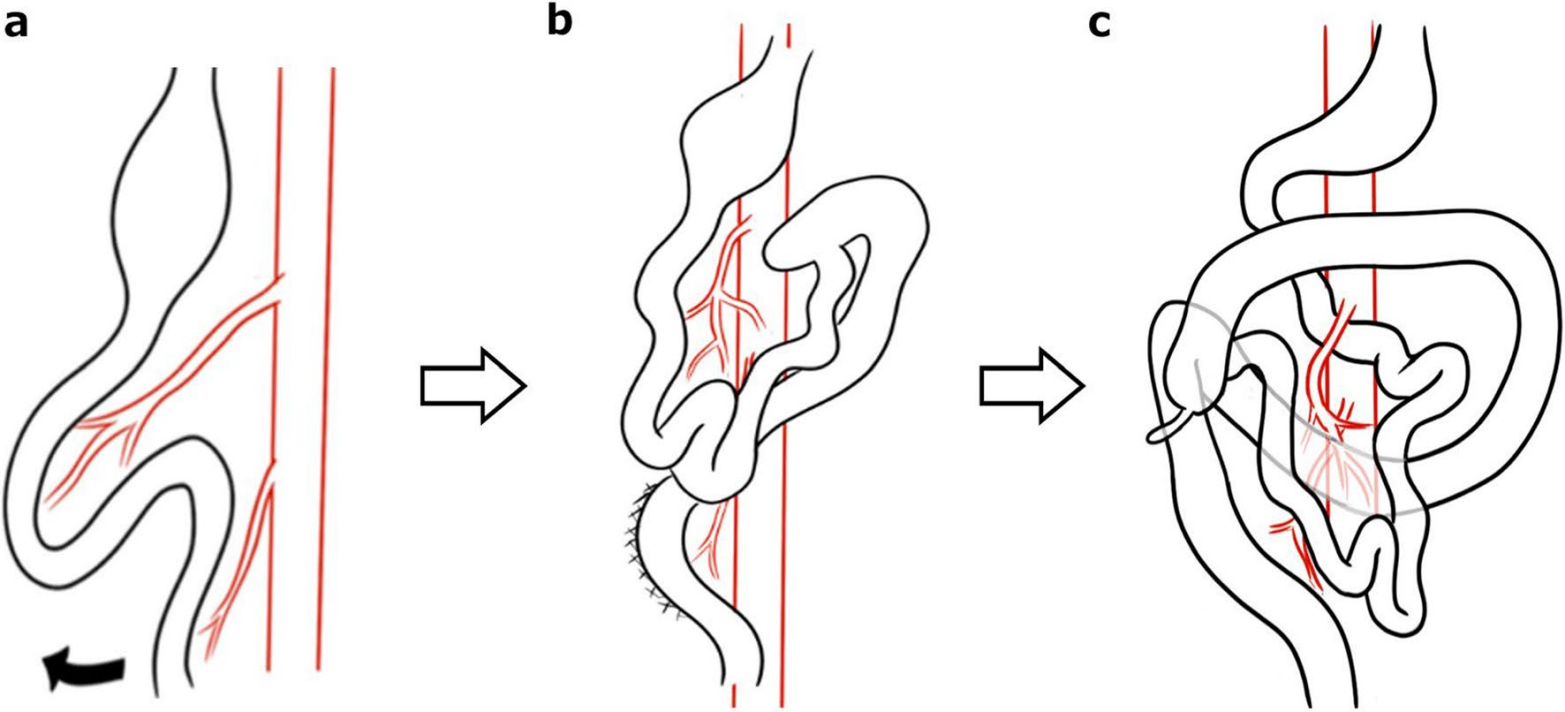
The developmental mechanism in this patient. The 90° rotation was incomplete or reversed in stage 1 (**a**), and the hindgut was fixed on the right side (**b**), followed by normal development in stages 2 and 3, where 270° counterclockwise rotation occurred and the right sigmoid colon was fixed in the form of a cecum covering the ventral side of the sigmoid colon (**c**)

In this case, the sigmoid colon was fixed to the right retroperitoneum, and the cecum was on its ventral side. The developmental mechanism of this case is thought to be that 90° rotation was incomplete or reversed in stage 1, and the hindgut was fixed on the right side, followed by normal development in stages 2 and 3, where 270° counterclockwise rotation occurred and the right sigmoid colon was fixed in the form of a cecum covering the ventral side of the sigmoid colon (Fig. 5).

Endoscopic treatment is the first choice for colonic LSTs because of their shallow depth, despite their large tumor diameter, and because of the low biological risk of submucosal invasion, especially for homogeneous LSTs [12]. In this case, endoscopic treatment was initially performed, but was difficult to complete. Therefore, the patient was also treated by laparoscopic surgery. As a possible explanation, we hypothesize that the sigmoid colon, which originally had a relatively high degree of freedom within the abdominal cavity, was completely fixed to the right side in this patient, and the hepatic flexure and descending colon were also fixed to the retroperitoneum, resulting in loss of freedom in endoscopic operations.

Only 19 cases of sigmoid colon with symmetrical IMA running and fixed on the right side have been reported [5, 10, 13–26], 11 of which were discovered during an anatomical evaluation or autopsy. There were 11 patients who had right-sided colon fixation only and 8 patients in whom the sigmoid colon was fixed dorsal to the cecum. After excluding patients who were discovered during anatomy or autopsy, 8 patients who were clinically diagnosed with fixation of the sigmoid colon dorsal to the cecum are summarized in Table 1 [5, 10, 15, 23–26]. An intraoperative diagnosis was made in 3 patients, and among the 5 patients who received a preoperative diagnosis, 3 were diagnosed by CECT and 2 were diagnosed by X-ray. Among the 4 patients in whom laparoscopic surgery was initiated, 3 were converted to laparotomy due to difficult intra-abdominal anatomy or adhesions. In 1 case, because it was difficult to detach the adhesion between the sigmoid colon and the liver at the hepatic flexure, and in two cases, because the abdominal cavity was observed by laparoscopy but was not a normal anatomy, the patients were converted to laparotomy.

**Table 1:** Reports of fixation of the sigmoid colon dorsal to the cecum

Author	Year	Age	Sex	Diagnostic procedure	Morphology of the sigmoid colon	Preoperative diagnosis of intestinal malrotation	Treatment
Pyrtek LJ	1960	36	M	X-ray	Posterior and lateral to the cecum	Yes	Laparotomy
		58	M	X-ray	Posterior and lateral to the cecum	No	Laparotomy
Choi NG	2013	71	M	Surgery	Posterior and lateral to the cecum	No	Laparoscopy → laparotomy
Kar H	2016	56	M	Surgery	Posterior and lateral to the cecum	No	Laparotomy
Singh M	2018	62	M	CECT	Posterior and lateral to the cecum	Yes	Conservative treatment
Watanabe K	2021	82	F	Surgery	Only right side	No	Laparoscopy
Bertelli G	2022	65	M	CECT	Posterior and lateral to the cecum	Yes	Laparoscopy → laparotomy
Lyu LJ	2022	56	M	CECT	Posterior and lateral to the cecum	Yes	Laparoscopy → laparotomy

**Fig. 6:**
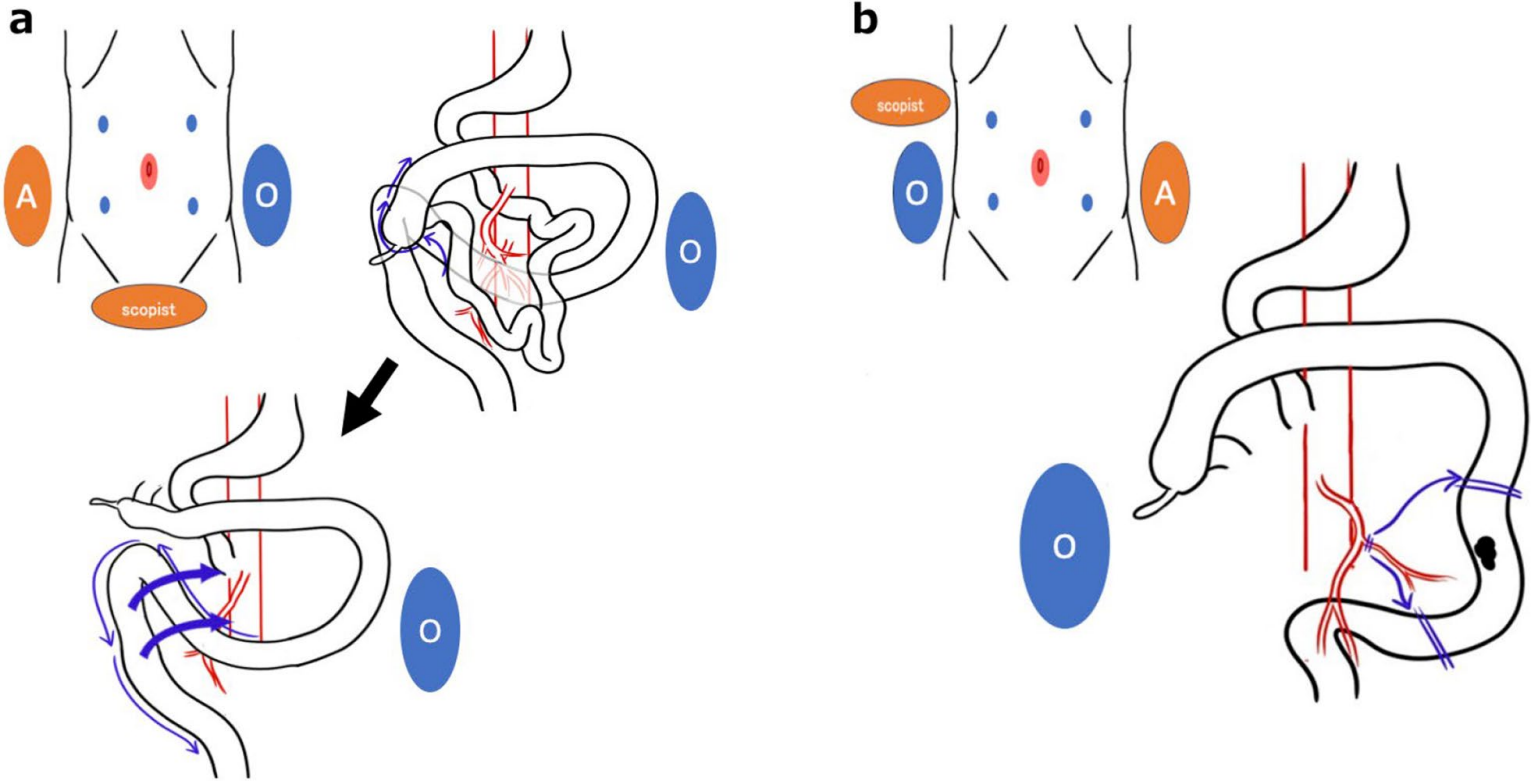
Surgical strategy. **a** The operator (O) and assistant (A) switched their standing positions from the positions that are usually adopted in laparoscopic surgery for the sigmoid colon, with the operator standing on the left side of the patient to start the surgery. Laparoscopic ileocecum and sigmoid colon mobilization was performed from the left side of the patient. **b** After the completion of mobilization, which returned the sigmoid colon and descending colon to anatomical normalcy, laparoscopic partial resection of the descending colon was performed

In this case, the sigmoid colon presented a symmetrical anatomy and was located on the right and dorsal sides of the ileocecum. The operator and assistant switched their standing positions from the positions that are usually adopted in laparoscopic surgery for the sigmoid colon, with the operator standing on the left side of the patient to start the surgery. The ileocecum mobilization was performed and the run of the sigmoid colon was clarified, laparoscopic sigmoid colon mobilization was performed from the left side of the patient (Fig. 6A). After the completion of mobilization, which returned the sigmoid colon and descending colon to anatomical normalcy, laparoscopic partial resection of the descending colon was performed (Fig. 6B). CECT and an enema examination allowed for an accurate anatomic evaluation, which revealed the IMA running symmetrically and the sigmoid colon running on the dorsal right side of the cecum. Subsequently, a detailed preoperative surgical simulation enabled safe laparoscopic surgery. If the vascular or intestinal anatomy is unclear on preoperative examination, detailed preoperative imaging using CT angiography, 3D-CT, or CT colonography may be needed. Intestinal malrotation is accompanied by abnormal adhesions in the abdominal cavity [6], and it may be difficult to complete laparoscopically, but with accurate preoperative anatomical evaluation and by devising surgical procedure, it may be possible to safely complete laparoscopically.

## Conclusions

Abdominal surgery involving malrotation requires detailed preoperative imaging and surgical simulation due to the anatomical diversity of patients with this condition.

## Data Availability

Not applicable.

## Abbreviations

SMA: Superior mesenteric artery
IMA: Inferior mesenteric artery
LST: Laterally spreading tumor
ESD: Endoscopic submucosal dissection
CECT: Contrast-enhanced computed tomography
POD: Postoperative day
